# Antimicrobial Resistance in the Context of Animal Production and Meat Products in Poland—A Critical Review and Future Perspective

**DOI:** 10.3390/pathogens13121123

**Published:** 2024-12-19

**Authors:** Patryk Wiśniewski, Miłosz Trymers, Wioleta Chajęcka-Wierzchowska, Katarzyna Tkacz, Anna Zadernowska, Monika Modzelewska-Kapituła

**Affiliations:** Department of Food Microbiology, Meat Technology and Chemistry, Faculty of Food Science, University of Warmia and Mazury, Plac Cieszyński 1, 10-726 Olsztyn, Poland; milosz.trymers@student.uwm.edu.pl (M.T.); wioleta.chajecka@uwm.edu.pl (W.C.-W.); ktkacz@uwm.edu.pl (K.T.); anna.zadernowska@uwm.edu.pl (A.Z.); monika.modzelewska@uwm.edu.pl (M.M.-K.)

**Keywords:** meat contamination, foodborne pathogens, meat safety, antibiotic, AMR

## Abstract

The prevalence of antimicrobial-resistant bacteria in meat and meat products is a significant public health challenge, largely driven by the excessive and inappropriate use of antimicrobials in animal husbandry. In Poland, a key meat producer in Europe, antibiotic-resistant pathogens such as *Campylobacter* spp., *Staphylococcus* spp., *Enterococcus* spp., *Listeria monocytogenes*, and Enterobacterales have been detected in meat, posing serious risks to consumers. This review examines the use of antimicrobial agents in meat production and the resulting antimicrobial resistance (AMR) in microorganisms isolated from meat products in Poland. The mechanisms of AMR, genetic factors, and prevalence in Poland are presented. It highlights key factors contributing to AMR, such as antibiotic misuse in livestock farming, and discusses the legal regulations governing veterinary drug residues in food. This review emphasizes the importance of monitoring and enforcement to safeguard public health and calls for further research on AMR in the meat industry. Antimicrobial resistance in meat and meat products in Poland is a huge challenge, requiring stricter antibiotic controls in animal husbandry and improved surveillance systems. Additionally, the impact of husbandry practices on the environment and food requires further research. Future efforts should focus on nationwide monitoring, alternative strategies to reduce antibiotic use, and stronger enforcement to combat antimicrobial resistance and protect public health.

## 1. Introduction

Antibiotic resistance is one of the most serious threats to public health worldwide [1,2]. The development and spread of antibiotic resistance are influenced by a variety of factors, including the misuse and overuse of antibiotics in human medicine, environmental contamination, and agricultural practices. Among these, the use of antibiotics in animal husbandry and meat contamination plays a particularly significant role, as resistant bacteria and resistance genes can infiltrate the food chain and impact human health [3,4]. It is increasingly shown that one of the key factors leading to this phenomenon is the failure to comply with regulations on the use of antibiotics in animal husbandry (in animals raised for food) [1]. Exceeding permitted doses, the inappropriate use of antibiotics for disease prevention in healthy animals, and the use of drugs critical for the treatment of human infections in livestock farming contribute to the selection of resistant bacteria that can infiltrate the food chain and pose a threat to consumers [1,2]. Despite the introduction of regulations and recommendations to limit antibiotic use in the animal husbandry sector, significant compliance gaps remain, making it difficult to effectively combat the spread of antibiotic resistance [5].

Antibiotic-resistant microorganisms represent one of the most serious challenges of modern medicine and agriculture [6]. The World Health Organization (WHO) has identified antimicrobial resistance (AMR) as a global health and food security threat, emphasizing the need for a “One Health” approach that integrates human, animal, and environmental health strategies [3]. The ability of bacteria to develop resistance to antimicrobial drugs is becoming increasingly widespread, and it affects not only the treatment of infections in humans but also the food sector [3]. Meat and meat products can be a reservoir of antibiotic-resistant pathogens, raising concerns for public health and the effectiveness of treating infectious diseases [3]. As one of the important meat producers in Europe, Poland faces the challenge of monitoring and controlling microbial resistance in the meat sector [7]. Resistance to drugs such as ampicillin, tetracycline, or gentamicin has been observed in numerous bacterial isolates (*Escherichia coli*, *Staphylococcus* spp., *Enterococcus* spp., *Klebsiella pneumoniae*, and *Citrobacter* spp.) [7].

The main factors contributing to the spread of antimicrobial resistance in foods of animal origin, with a particular focus on meat and meat products, are the inappropriate and excessive use of antimicrobials [8,9]. In practice, about 80% of globally produced antibiotics are used in animal production; however, some that are classified as antibiotics have other purposes in animal production than for treating diseases. Some farmers use subtherapeutic doses of antibiotics to obtain various aims such as animal growth increase, weight gain acceleration, digestion improvement, a higher feed conversion ratio (FCR) and to prevent or reduce disease outbreaks [10]. Residues of veterinary medicines may be present in food of animal origin (ASF) even if their use is fully regulated by law [11,12]. However, some farmers do not pay sufficient attention to withdrawal periods (WDPs) which increases the risk of spreading antimicrobial resistance in food worldwide, especially in developing countries [13].

The European Medicines Agency (EMA) defines a Maximum Residue Limit (MRL) as an acceptable concentration of residues in food products, and the European Union requires that foods do not contain residues of veterinary medicines above the MRL. The European Union (EU) legally requires that foods like meat, milk, or eggs not contain residue levels of veterinary medicines or biocidal products that could endanger the consumer’s health. Regulation (EC) No 470/2009 of the European Parliament and of the Council [14] defines rules for setting maximum permissible levels (MRLs), measured in milligrams per kilogram for solid products and milligrams per liter for liquids [15,16]. Antibiotics can accumulate in tissues such as muscles and organs, and their residues act as selection factors that promote the development of resistance in the microorganisms present [8]. Antibiotic residues in muscles post-mortem represent a selection stress that allows only those bacteria with appropriate resistance mechanisms (including enzymatic degradation of the antibiotic, modification of the antibiotic’s target site, or active removal of the substance from the cell) to survive [17]. Such strains not only survive but can also transfer resistance genes to other bacteria through a process of horizontal gene transfer [18]. This requires the interaction of regulatory authorities in monitoring and enforcement and using accurate analytical methods to detect AMR in meat products [10].

The use of antimicrobials in animal husbandry is inevitable [10]. AMR bacteria are frequently detected in meat and meat products, which results from the use of antibiotics during the treatment of sick animals or the preventive treatment of healthy ones [10]. Among pharmaceutical residues, the most common are antibiotics and anthelmintic agents, with antibiotics being the most extensively used in both human and veterinary medicine [10]. Due to health concerns, antibiotics for food preservation have been banned in many countries [10,19,20,21,22].

This review aims to critically discuss the available literature, based on an expert analysis of the topic of antimicrobial resistance in microorganisms isolated from meat and meat products in Poland, as well as the use of antimicrobial agents in animal production in this country. The review focuses on the main factors contributing to the spreading of antibiotic resistance, such as the excessive and improper use of antimicrobial agents in animal husbandry. It also discusses the legal regulations regarding veterinary drug residues in animal-derived food products, as well as the importance of monitoring and enforcing these regulations to protect public health. The study aims to highlight the risks associated with antimicrobial resistance in meat and meat products and the need for further research and monitoring in this area.

## 2. Antibiotics Used in Animal Production

### 2.1. Importance of Antibiotic Use in Livestock Production

Animal husbandry is of considerable importance in agriculture in countries of the European Union. Obtaining the best results from animal husbandry depends primarily on the use of high-quality feed [23]. Ensuring the free circulation of safe and valuable food and feed products is a key element of the internal market, which has a significant impact on consumer health and satisfaction [23]. The use of antibiotics is inextricably linked to obtaining the best results from animal husbandry [24]. Most of the residues of these agents are found in various food products—both of animal and plant origin [24]. Humans can come into contact with antibiotics from two main sources: firstly, from medicines prescribed by doctors, and secondly, from substances used in animal husbandry [25]. These antibiotics can cause serious health problems in humans, which has prompted the introduction of maximum residue limits in food safety legislation. The most important factor contributing to the presence of antibiotics in food is their overuse (including overdosing and ignoring the withdrawal period), as well as the use of antibiotic-contaminated water and improper disposal of animal manure [21].

The use of antibiotics in animal feed for growth promotion became more prominent in the 1950s and 1960s, when various antibiotics with different mechanisms of action were introduced into animal feed. Supplementation of animal feed with antibiotics and antibiotic growth promoters (AGPs) continued until public health concerns arose about off-target drug levels in meat and animal products, increased antimicrobial resistance, intestinal dysbiosis, etc. [26]. Based on the results of studies showing an increase in the number of resistant bacteria under the influence of the cessation of AGP use in various countries, the European Union banned the use of antibiotic growth promoters in all Member States as of 1 January 2006 (Regulation (EC) No 1831/2003) [26]. As of that year, antibiotics in animal husbandry must be used for therapeutic purposes.

The cost of producing medicated feed is high, and meeting veterinary requirements is difficult for small- and medium-sized farms, which can lead to non-compliance [27]. Pharmaceutical and veterinary control often lack the tools to prevent illegal trade in veterinary medicines [27]. A monitoring carried out in Poland showed that antibiotics were used in animal farms, especially on turkey and broiler farms. The monitoring results indicated legitimate concerns about the impact on public health now and in the future [28].

### 2.2. Challenges of Antibiotic Use

The main purpose of antimicrobial use is to control and treat bacterial infections. Antibiotics are administered to symptomatic animals, and the agent dose is adjusted according to their condition. Among farm animals, individual treatment is used for dairy cows and calves [29]. It should be noted that such treatment is ineffective for animals in large flocks, e.g., more than 30,000 poultry or 100 piglets [29]. Antimicrobials are administered to the whole herd for large groups of animals when individual animals show signs of disease. This is known as metaphylaxis [29]. Early treatment of the entire herd reduces the number of sick or dead animals and lowers the use of antibiotics, resulting in lower treatment costs [29]. The prophylactic use of antibiotics is a way of preventing possible infections to which animals are exposed [30]. In this case, agents are administered to individuals or the entire herd when there are no clinical signs of disease, but there is a high probability of infection [30]. Antibiotics are also administered prophylactically at so-called critical moments for the animals, e.g., when mixing animals from different herds, transport, or at the end of lactation of dairy cows [30,31]. AGPs were another way of using antibiotics in animal production [30]. However, the use of antimicrobial substances in animal husbandry was banned by law in 2006 [30]. The effect of growth promoters was not only to increase weight gain (by 4–28%) but also to improve nutrient absorption, leading to more efficient feed conversion (by 0.8–7.6%) [30]. In addition, there were also reductions in methane and ammonia emissions and more efficient phosphorus utilization [30]. In addition, the use of AGPs reduced the number of sick animals and livestock losses [32]. The use of such agents prevented gastrointestinal infections and maintained the balance of the intestinal microflora [30,32].

### 2.3. Antibiotic Use in Poland

The use of antibiotics in livestock production is a globally important issue, and the challenges of monitoring and reducing their use have been repeatedly highlighted in the literature. Pyzik et al. [33] note the lack of global reporting systems for antibiotic use and call for mandatory reporting in every country, not just in Europe. There is also a need to implement monitoring procedures, more effective biosecurity, better governance, and educational efforts targeting groups such as food producers and growers to raise awareness of the risks of antibiotic use.

In Poland, as the report of the Supreme Chamber of Control (NIK) [28] indicates, the use of antibiotics in livestock production is widespread, and supervision proves ineffective. For example, in the Lubuskie Voivodeship, as many as 70% of farmers on monitored farms used antibiotics, always justifying their use for therapeutic reasons. However, the NIK points to the lack of full documentation of treatment and weaknesses in the surveillance system, which often relies on breeders’ statements. The scale of the use of antibiotics remains unknown, although data show a 23% increase in their sale between 2011 and 2015. The NIK recommends making reporting mandatory, creating a nationwide database and implementing educational programs for breeders to better control the situation and counter antibiotic resistance.

A report by the European Medicines Agency (EMA) [34] shows that although Poland has seen a decline in sales of veterinary antibiotics, their use per kilogram of body weight of production animals still exceeds the EU average. The most-used classes of antibiotics in Poland are tetracyclines, penicillins, and sulfonamides, and the use of critically important antibiotics for human medicine has been limited. Programs being implemented, such as the National Program for the Protection of Antibiotics, aim to rationalize their use and educate farmers and veterinarians. Despite progress, continuing to reduce the use of these agents, especially those critical to human health, remains a challenge.

The World Health Organization (WHO) [35] reports that some 27 different antimicrobials are used in animals, including critically important macrolides, ketolides, glycopeptides, quinolones, polymyxins, and cephalosporins (third and fourth generation) for human medicine. The lack of a global surveillance system for the use of antibiotics in the livestock sector is a major gap. In human medicine, the Global Antimicrobial Surveillance System (GLASS) [3] has been implemented to collect and analyze antibiotic resistance data. An analogous system is lacking in the animal sector, although the Scandinavian countries that have implemented advanced monitoring systems can serve as an example of good practice. In low- and middle-income countries, this surveillance is only just developing, with global resistance trends mapped mainly by point prevalence surveys [1].

Studies have shown that between 2000 and 2018, resistance levels increased in chickens and pigs, while stabilizing in cattle, with significant geographic differences [1]. These data underscore the urgent need for global action to reduce antibiotic use in animal husbandry, implement more effective surveillance mechanisms, and promote the rational use of antimicrobials in animal production.

## 3. Influence of Food Processing Technology on the Antibiotic Content in Meat Products

Modern consumers pay attention to the health-promoting properties of food. Meat and meat products are perceived as a source of protein, vitamins, and minerals [36]. Meat is also a source of bioactive compounds such as L-carnitine, taurine, anserine, carnosine, coenzyme Q10, glutathione, bioactive peptides, isomers of linoleic acid (CLA), creatin, and haem iron [36]. In addition to compounds essential for supporting human health, meat may contain drug residues. They result from the inappropriate use of veterinary medicines and the failure to comply with the withdrawal period [37]. This, in turn, can significantly reduce the quality and safety of meat and meat products, which is a major challenge in the context of producing healthy and safe food [37]. Most raw materials of animal origin undergo heat treatment or other processing methods before being consumed. The purpose of these is, among other things, to increase digestibility, improve sensory properties and ensure food safety—by eliminating pathogens [38]. Heat treatment of meat also reduces the concentration of drug residues (Table 1) through protein denaturation, loss of water and fat, and a change in pH [39,40,41,42,43,44,45,46]. For example, the concentration of doxycycline in meat decreases during cooking, and the residues are excreted from the muscle with cooking loss [39].

Different food processing techniques affect changes in antibiotic content (degree of reduction) in various ways, which include the type and parameters of processing, the kind of meat, the type of antibiotic, or the initial antibiotic content (Table 1). Boiling proved to be one of the most effective methods of heat treatment. For poultry boiled at 100 °C for 5 min, the enrofloxacin (ENO) concentration decreased from 746.34 ± 5.62 μg/kg to 237.53 ± 2.13 μg/kg, representing a 68.17% reduction [46]. Similarly, oxytetracycline (OTC) decreased from 824.16 ± 7.20 μg/kg to 383.33 ± 3.70 μg/kg (53.49% reduction), and ciprofloxacin (CIP) dropped from 643.14 ± 6.97 μg/kg to 205.46 ± 9.72 μg/kg, achieving a 68.05% reduction. Prolonged boiling, such as for 15 min, resulted in even greater decreases in antibiotic content. For instance, OTC in pork showed a reduction of 52.69%, with the concentration decreasing to 236.56 ± 7.96 μg/kg [42]. Sulfonamides, including sulfadiazine (SDZ), sulfamethoxazole (SMX), sulfamonomethoxine (SMM), and sulfaquinoxaline (SQ), demonstrated gradual reductions in concentration with extended boiling times. For example, SDZ in poultry boiled at 100 °C for 3 min showed a 40.48% reduction, while a 12 min boiling time resulted in a 60.71% reduction [44].

Roasting was another processing method analyzed. Roasting poultry at 200 °C for 30 min reduced the ENO concentration from 746.34 ± 5.62 μg/kg to 233.23 ± 10.19 μg/kg, corresponding to a 68.75% reduction [46]. Similarly, CIP levels dropped from 643.14 ± 6.97 μg/kg to 200.98 ± 10.02 μg/kg, also achieving a 68.75% reduction. However, roasting at lower temperatures (170 °C) for varying durations was less effective in reducing sulfonamide levels. For instance, roasting for 6 min reduced SQ by 21.66%, while roasting for 12 min achieved a 37.73% reduction.

Microwave cooking showed high effectiveness, particularly at higher power levels and longer cooking times. Cooking poultry in a microwave at 900 W for 3 min reduced OTC levels from 824.16 ± 7.20 μg/kg to 227.67 ± 2.10 μg/kg, corresponding to a 72.38% reduction [46]. CIP levels decreased by 55.16%, reaching 288.40 ± 3.23 μg/kg. Shorter microwave times and lower power settings (440 W for 45 s) were less effective but still resulted in notable reductions. For instance, tetracycline (TET) levels in poultry decreased by 59.89%, while in pork, the reduction reached 80.54% [40]. The data suggest a clear correlation between the intensity of microwave processing and the effectiveness of antibiotic reduction.

Grilling, despite utilizing high temperatures, was less effective than other methods. For poultry grilled at 8 kW for 2.5 min, ENO levels decreased by only 33.33%, while OTC levels dropped by just 16.67% [46]. Reductions for CIP and doxycycline (DOX) were similarly modest, at approximately 16.66–16.67%. This suggests that the short duration of grilling, combined with high intensity, resulted in less degradation of antibiotic residues compared to longer and more evenly distributed heating processes.

The analysis of the data indicates that the effectiveness of antibiotic reduction in meat depends on the processing method, the duration of the process, and the type of antibiotic. Boiling and microwave cooking were the most effective methods, with longer durations and higher intensities achieving reductions of over 70%. Roasting and grilling, despite employing high temperatures, were less effective, particularly for shorter durations. Additionally, studies reveal that while thermal processing reduces antibiotic residues, it may lead to the formation of degradation products with potential health implications. For example, Gratacós-Cubarsí et al. [40] observed that tetracyclines in poultry and pork degrade under heat, forming anhydrotetracyclines, which retain some biological activity. Nguyen et al. [42] highlighted the toxic potential of oxytetracycline degradation products in animal models, and Furusawa and Hanabusa [44] found that cooking significantly reduces sulfonamide levels, though complete elimination remains challenging. These findings emphasize the dual role of food processing in reducing antibiotics and potentially generating bioactive or toxic degradation products, underlining the need for further research to optimize processing techniques and assess their implications for consumer safety.

The presence of drug residues in meat might cause a serious problem in the production of fermented meat products since the components of industrial starter cultures for fermented meat products might be susceptible to antibiotic residues. In this case, a fermentation process might be disrupted or altered, which not only results in obtaining meat products with changed sensory properties but also poses a risk to public health. Previous studies by Darwish et al. [47] and Moyane et al. [48] showed that the altered fermentation process caused an outbreak of foodborne illness as pathogens present in the raw material persisted after poor fermentation. According to a study by Kjeldgaarda et al. [49], it appears that the permitted levels of antibiotics in meat can negatively affect the fermentation process. They showed that bacteria used as starter cultures are susceptible to antibiotic residues, even at levels close to those allowed by law, which can lead to the presence of pathogens in processed sausages. Their findings suggest that such residues could be the cause of disease outbreaks associated with the consumption of fermented meat products, providing an argument for reducing the use of antibiotics in animal husbandry [49].

Studies presented here show that the choice of heat treatment method plays a key role in reducing antibiotic residues in meat products, which is directly relevant to food safety and public health.

## 4. The Problem of Antibiotic Resistance

Antibiotic resistance among pathogenic bacteria increases morbidity and mortality and is therefore a challenge worldwide [50]. Of particular concern is the emergence of multidrug resistance [50,51,52]. The scale of antibiotic-resistant bacteria in the environment of animal farms observed worldwide today is a consequence of the widespread use of antibiotics at least a decade earlier [27]. Very often, the same antibiotics that were used in agriculture and veterinary medicine were also used to treat humans. For therapeutic purposes, they should only be administered to animals with a confirmed infection [27]. However, it is common practice to administer antibiotics to the whole herd by giving prophylactic doses of antibiotics in poultry, cattle, and pig farming, which are much higher than those used for therapeutic purposes [27]. The Chief Veterinary Inspectorate has been monitoring the drug resistance of zoonotic bacteria in Poland since 2014, and the results show an increase in the drug resistance of microorganisms. Intensive agriculture has a high level of pollutants emitted into the environment, such as air, soil, surface, and rainwater [53,54,55]. The use of manure as a fertilizer carries the risk of environmental contamination by pathogens, antibiotics, and antibiotic-resistant pathogens [52].

**Figure 1:**
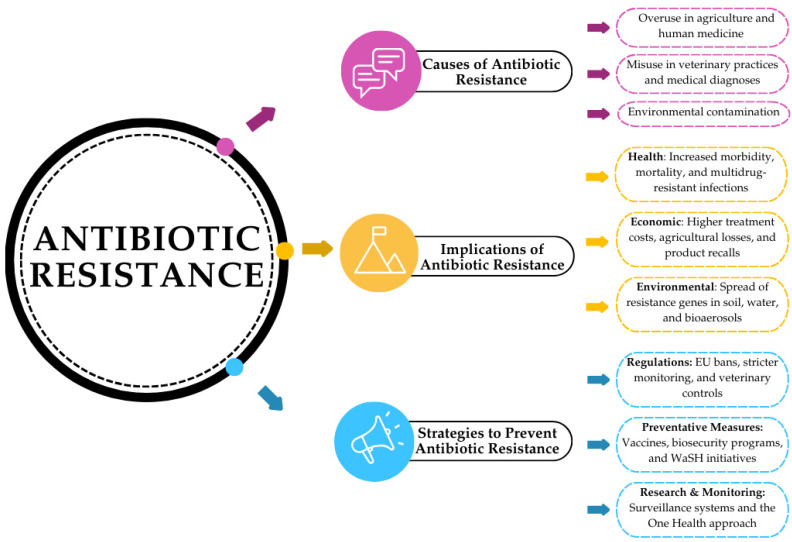
The problem of antibiotic resistance.

Figure 1 summarizes the main causes of antibiotic resistance, such as overuse of antibiotics in agriculture, poor veterinary practices, and environmental pollution. It also outlines the health, economic, and environmental implications of resistance, and emphasizes the importance of regulation and preventive measures such as bioassurance programs, vaccination, and One Health approach initiatives.

### 4.1. Regulations in Antibiotic Use

The European Medicines Agency sets maximum residue limits and requires that food not contain harmful amounts of veterinary medicines. Illegal practices, such as off-label use of approved drugs, also contribute to the problem [56]. The use of antibiotics in veterinary medicine has been uncontrolled, but legislation is now being introduced to regulate the practice [56]. However, it is difficult to assess practice on animal farms in Poland due to inconsistencies between reports of antibiotic use and the surveillance system for these drugs [57].

In Poland, one of the laws regulating medicinal products, including antibiotics, is the Act of 6 September 2001 on Pharmaceutical Law. It defines the use of medicinal products in humans and animals, establishes rules for the production and authorization of medicines, and regulates the conduct of clinical trials [58,59]. The Act of 11 March 2004 on the protection of animal health and the control of infectious animal diseases imposes an obligation on veterinarians to keep veterinary medical records of the treatment carried out. Regulation (EU) 2019/6 of the European Parliament and of the Council of 11 December 2018 on veterinary medicinal products [25], repealing Directive 2001/82/EC, defines the use of antimicrobials in the treatment of animal diseases. The provisions of this regulation entered into force on 28 January 2022. It introduces important requirements for medicinal products for use in animals, aiming to improve public health and animal health, and reduce antibiotic resistance. Most notably, it bans the prophylactic use of antimicrobials in healthy animals (except in exceptional cases), places restrictions on the use of antibiotics important for human treatment, and requires detailed monitoring and reporting of their sale and use. It sets stricter conditions for registration and introduces a single authorization system in the EU market to increase the quality, safety, and availability of medicinal products. Only veterinarians can prescribe medicines for animals, limiting independent use by pet owners. The regulation also promotes research into new, safe medicinal products and tightens import rules to ensure they comply with EU standards. All these regulations are part of the European Union’s strategy for health safety and the fight against antimicrobial resistance [25]. However, none of the above legal requirements prohibit the therapeutic use of antimicrobial substances but only restrict their unjustified use [30].

In the European Union, since January 2006, following Regulation No 1831/2003 of the European Parliament and of the Council of 22 August 2003 [23], the marketing and use of antibiotics as feed additives have been prohibited. In Poland, veterinarians providing veterinary services are responsible for keeping drug circulation records and veterinary documentation, including prescription medicinal products for use in both livestock and pets [27]. Currently, the use of antibiotics for growth promotion in farm animals and poultry is banned throughout the EU. However, this ban has not significantly reduced the use of antimicrobials, and subtherapeutic use has been replaced by metaphylaxis and prophylaxis [52,53].

### 4.2. Implications of Antibiotic Resistance

Antibiotic resistance leads to higher rates of morbidity and mortality, particularly because of infections with multidrug-resistant bacteria [60]. These bacteria are more difficult to treat, resulting in longer hospital stays and an increased risk of complications and deaths [60]. Antibiotic resistance in Poland leads to serious health risks. Another problem is global bacterial resistance, which can lead to ineffective standard antibiotic therapies and higher hospital admissions [61]. The costs associated with antibiotic resistance are enormous, both for healthcare systems and the economy. Inappropriate use of antibiotics in Poland, especially in primary care, leads to high treatment costs for infections caused by resistant bacterial strains. Research shows that the overuse of antibiotics in regions with high levels of unemployment and intensive population mobility contributes to increased resistance and economic burden, including prolonged hospitalization and higher treatment expenditure [62].

The costs associated with treating infections caused by resistant bacteria from food are significant [62]. High levels of antibiotic resistance, especially in egg products, affect consumer health, leading to increased healthcare expenditure, including longer hospital stays and the cost of additional diagnostic tests and treatment [63]. In an economic context, bacterial resistance in the agricultural sector in Poland also leads to losses in agricultural production, as animals infected with resistant bacteria require more complex treatment, which increases the cost of breeding [62,63]. These costs also include losses associated with product recalls and the costs of monitoring and controlling infections in agricultural production [64]. Combating antibiotic resistance in the food production sector is a complex process that requires cooperation at local and national levels. These costs also extend to the agricultural sector, where the use of antibiotics in animal husbandry leads to production losses due to increasing drug resistance in both humans and animals [64]. Research indicates that vaccines can be an economically viable tool in the fight against antibiotic resistance, reducing the number of cases of resistant infections and reducing the overall need for antibiotics [64].

Antibiotic resistance also has a significant impact on the environment. The use of antibiotics in agriculture and animal husbandry leads to contamination of soil and water, which promotes the spread of resistance genes in the environment [65]. Excessive use of antibiotics in animal husbandry and poor waste management lead to antibiotics and resistant bacteria entering the environment, including soil and groundwater [65]. Studies on isolated strains from food products indicate that resistant bacteria can infiltrate the ecosystem through agricultural and industrial waste, increasing the risk of resistance genes spreading in the environment [65].

Antibiotic resistance in Poland, associated with isolated bacteria from food, is a serious health, economic, and environmental threat. Effective measures are needed to reduce the use of antibiotics in food production and to monitor the spread of resistance.

### 4.3. Strategies to Prevent Antibiotic Resistance

There is a need to integrate water, sanitation, and hygiene (WaSH) programs with biosecurity in animal husbandry. This approach can reduce the transmission of antibiotic-resistant bacteria [66]. Biosequestration and improved hygiene in animal husbandry can significantly reduce the risk of exposure to resistant bacteria, protecting both humans and animals [66]. The One Health approach emphasizes the importance of the interdependence between human, animal, and environmental health [67]. The implementation of integrated measures, such as reducing the overuse of antibiotics and improving sanitation and hygiene in animal husbandry, are key actions in the fight against antibiotic resistance [67]. These programs should be combined with better monitoring and surveillance systems to effectively prevent the further spread of resistant bacteria [67]. Intensive animal husbandry in Poland results in the emission of bioaerosols containing antibiotic-resistant bacteria. These bacteria can enter the environment, threatening the health of humans and animals in the vicinity of farms [27]. Action is needed to reduce the spread of antibiotic-resistant bacteria on farms and in the animal food supply chain [27].

In Poland, monitoring and surveillance of the spread of antibiotic-resistant bacteria in the agricultural environment is insufficient [27]. Studies to date show the presence of antibiotic-resistant bacteria on farms in Poland, but data are limited to individual farms and a small number of samples [27]. Larger surveys and more extensive monitoring programs are needed to better assess the scale of the problem [27].

## 5. Antibiotic Resistance Among Microorganisms Isolated from Meat and Meat Products

Bacteria such as *Campylobacter* spp., *Staphylococcus* spp., *Enterococcus* spp., *Listeria monocytogenes*, and Enterobacterales (including *Salmonella* spp. and *E. coli*) are found in the animal farm environment and are emitted into the air and surface water, which can cause infections in humans and are a source of antibiotic resistance genes [52]. Many bacteria have evolved multiple mechanisms of antibiotic resistance, including the production of inactivating enzymes, blockade of target sites, alteration in cell membrane permeability, and active efflux of antibiotics from the cell [68]. Bacteria may have resistance genes for many different drugs, as well as transport proteins that can actively pump drugs and substances out of the cell into the external environment [68]. Table 2 presents the occurrence and antimicrobial resistance of microorganisms isolated from meat and meat products in Poland.

### 5.1. Campylobacter spp.

*Campylobacter* spp. is a major cause of foodborne illness in humans, which results from improper processing or consumption of undercooked poultry meat [97]. For severe or chronic infections caused by *Campylobacter* spp., treatment with antibiotics (e.g., fluoroquinolones and macrolides) may be necessary, which is problematic because of the uncontrolled use of these drugs in clinical medicine and animal production [98].

*Campylobacter* spp. is one of the main causes of foodborne gastroenteritis responsible for zoonosis—campylobacteriosis. *Campylobacter*, especially *Campylobacter jejuni* and to a lesser extent *Campylobacter coli*, is one of the leading causes of foodborne infections worldwide [99,100]. The main source of infection is contaminated poultry meat [101], and high contamination poses a threat to public health. It is estimated that 50% to 80% of human campylobacteriosis cases are directly linked to poultry meat, particularly *Campylobacter jejuni* [102,103]. In recent years, *Campylobacter* has been increasing in resistance to antibiotics (especially quinolones and macrolides) due to their widespread use in agriculture [72,104]. Although campylobacteriosis usually resolves spontaneously, macrolides (erythromycin), fluoroquinolones, and tetracyclines are used in severe cases [105].

Since chickens are the main reservoir of *Campylobacter*, antibiotic resistance in these bacteria isolated from poultry is of serious concern. The use of antimicrobials in animal production, especially in veterinary medicine, may contribute to the buildup of resistance in human isolates, especially to quinolones [104,106]. The aim of the study by Woźniak-Biel et al. [74] was to identify *Campylobacter* strains, isolated from turkeys and chickens, using polymerase chain reaction (PCR) and matrix-assisted laser desorption/ionization time-of-flight (MALDI-TOF) methods, and assess their antibiotic resistance. The results obtained from MALDI-TOF were consistent with those from multiplex PCR. There was 100% resistance to ciprofloxacin in strains from turkeys and chickens, and 58.1% and 78.6% resistance to tetracycline in these groups, respectively. No multidrug-resistant strains were detected, and all ciprofloxacin-resistant strains had a mutation in the *gyrA* gene at the Thr-86 position. The presence of the *tetO* gene was present in 71.0% of turkey strains and 100% of chickens, and this gene was also found in five turkey strains and three chickens that were sensitive to tetracycline. The results indicate a high prevalence of *Campylobacter* strains that are phenotypically and genetically resistant to fluoroquinolones and tetracycline.

A study by Maćkiw et al. [76] on the antibiotic resistance of *C. jejuni* and *C. coli* strains isolated from food in Poland showed that *Campylobacter* spp. is often isolated from poultry, which is the main source of human infections with these bacteria. High levels of resistance to fluoroquinolones, including ciprofloxacin, were found, which is in line with trends observed in other European countries. Resistance to tetracyclines was also common, which may be due to the widespread use of these antibiotics in animal husbandry. The *tet*(O) genes responsible for resistance to tetracyclines and *gyrA* associated with resistance to fluoroquinolones were identified. Some strains showed resistance to macrolides such as erythromycin, but this was less prevalent compared to fluoroquinolones and tetracyclines. It was also noted that multidrug resistance was relatively common. These results suggest the need to monitor *Campylobacter* sp. resistance in food to prevent the spread of resistant strains, which can threaten public health.

A study by Wieczorek and Osek [73] analyzing the antibiotic resistance of *C. jejuni* and *C. coli* strains of poultry carcass samples collected between 2009 and 2013 showed that 54.4% of samples were positive for *Campylobacter*. Resistance to ciprofloxacin was 81.6%, to tetracycline 56.1%, and only 2.4% of isolates were resistant to erythromycin. In contrast, resistance was higher among *C. coli* than *C. jejuni*, and an increase in resistance to ciprofloxacin and tetracycline was noted over the five-year study period. A later study by Wieczorek et al. [72] on the prevalence and antibiotic resistance of *Campylobacter* strains isolated from chicken carcasses in Poland between 2014 and 2018 reported that 53.4% of samples (in total 2367 samples collected from slaughterhouses across the country) were positive for *Campylobacter*. Mainly, *C. coli* (31.2%) and *C. jejuni* (22.2%) were identified. The strains showed high resistance to ciprofloxacin (93.1%), nalidixic acid (92.3%), and tetracycline (70.9%). Only a small percentage of isolated strains were resistant to erythromycin (4.2%), with *C. coli* (6.4%) showing more resistance than *C. jejuni* (1.1%). Multidrug resistance was found in 25.1% of *C. coli* and 20.6% of *C. jejuni* strains. The study showed an increase in the percentage of multidrug-resistant strains compared to earlier years, indicating the necessity of taking measures to control *Campylobacter* at the poultry slaughter stage and restricting the use of antibiotics in poultry production.

Rożynek et al. [71] analyzed in detail the emergence of macrolide-resistant *Campylobacter* strains in poultry meat in Poland and the resistance mechanisms responsible for the problem. Macrolides, such as erythromycin, are key antibiotics used to treat infections caused by these bacteria [71]. The study found a significant number of strains resistant to macrolides, which poses an important therapeutic challenge. The mechanism of resistance to these antibiotics was mainly related to mutations in domain V of the 23S rRNA gene, which encodes the ribosomal subunit responsible for macrolide binding. These mutations, particularly at nucleotide positions 2074 and 2075, lead to a reduced ability of macrolides to inhibit bacterial protein synthesis [71]. Also identified were *erm*(B) genes that encode methyltransferases, enzymes that modify ribosomes and cause macrolide resistance. In addition, other resistance mechanisms, such as the pumping of antibiotics out of bacterial cells by the efflux pump CmeABC, were also identified as an important factor in the development of resistance. The study also found a link between resistance and intensive antibiotic use in poultry farming, which promotes the selection of resistant strains. The authors emphasize the need to monitor antibiotic resistance and to introduce stricter regulations on the use of macrolides in animal food production to prevent the further spread of resistant strains of *Campylobacter* spp.

Another source of *Campylobacter* is beef and pork. It was reported that the prevalence of *Campylobacter* spp. in retail beef products was about 10.0% [69,107], whereas its prevalence in beef and pork carcasses was 10.0% and 30.0%, respectively [108]. Antibiotic profiling revealed that *Campylobacter* isolated from pork and cattle carcasses during the slaughter process in Poland most often showed resistance to quinolones (57.1%) and tetracycline (51.4%) [70]. One strain of *C. coli* from a pork sample was resistant to three antibiotics simultaneously. This is worrisome given the public health concerns arising from the increasing antibiotic resistance of microorganisms to antimicrobials that are used as first-line drugs in the clinical treatment of campylobacteriosis [69]. As reported by Wieczorek and Osek [70], 100% of *Campylobacter* strains isolated from pork and beef carcasses were sensitive to gentamicin and chloramphenicol. Significant differences were found between *C. coli* and *C. jejuni*, especially in resistance to streptomycin (*p* < 0.001) and tetracycline (*p* < 0.05). All *C. jejuni* isolates were sensitive to streptomycin, while 80.5% and 66.7% of *C. coli* strains from pigs and cattle, respectively, were resistant. *C. coli* also showed higher resistance to tetracycline, quinolones (nalidixic acid), and fluoroquinolones (ciprofloxacin). Four *C. coli* isolates from pig carcasses were resistant to erythromycin. Multidrug resistance was found in 61.4% of strains, with the highest levels of resistance to quinolones, fluoroquinolones, aminoglycosides, and tetracyclines, mainly in *C. coli*.

*Campylobacter* spp. is also prevalent in geese and poses a potential risk for human campylobacteriosis through the consumption of goose meat. *Campylobacter* was found in 83.3% of goose cecum samples and 52.5% of neck skin samples from carcasses, with *C. jejuni* being the predominant species (87.7% of isolates) [75]. The isolates exhibited high levels of antimicrobial resistance, particularly to quinolones (90.8%) and tetracycline (79.8%), while resistance to macrolides was rare (0.6%) [75]. This aligns with findings from other studies showing high resistance of *Campylobacter* isolates to ciprofloxacin, tetracycline, and nalidixic acid in various bird species [68,109,110].

*Campylobacter* spp. in meat and meat products in Poland indicates the presence of this pathogen in both beef, pork, and poultry, with poultry meat being the main source of human infections. Studies have shown significant levels of antibiotic resistance, especially to quinolones and tetracycline, posing a serious public health challenge. Macrolide resistance, although rarer, is also a problem, especially in *C. coli*. *Campylobacter* strains, which have also shown multidrug resistance, underscoring the need for the close monitoring of antibiotic resistance and limiting the use of antibiotics in animal production. The increase in the number of multi-resistant strains in recent years poses an epidemiological threat and calls for action to control *Campylobacter* at all stages of food production.

### 5.2. Staphylococcus spp.

Antibiotic resistance in staphylococci isolated from meat and meat products has become an important public health problem worldwide [111]. Both coagulase-positive staphylococci (CPS) and coagulase-negative staphylococci (CNS) have been found to carry antibiotic-resistant genes, posing a potential threat to consumers [111,112]. Studies have shown a high prevalence of antibiotic-resistant *Staphylococcus* species in a variety of meat products, including chicken, beef, and processed meat products [112,113,114]. Interestingly, the distribution of antibiotic resistance varies by *Staphylococcus* species and meat type [80].

The pathogenesis of CNS species depends on the factors required for their commensal lifestyle, and one such factor that increases the importance of these microorganisms in the pathology of mammals and birds is their resistance to numerous antimicrobial agents [115]. Poultry has been identified as one of the most important carriers of foodborne pathogens and antimicrobial resistance genes [116]. A detailed analysis of resistance genes in staphylococci associated with livestock revealed a wide variety of these genes. These mainly include genes known to be commonly present in staphylococci of human and animal origin, such as the beta-lactamase gene *blaZ*, the methicillin resistance gene *mecA*, the tetracycline resistance genes *tet*(K), *tet*(L), *tet*(M), and *tet*(O), macrolysine–lincosamide–estreptogramin B (MLSB) resistance genes *erm*(A) and *erm*(B), erythromycin-inducible resistance gene *msr*A/B, *aac (6′) Ie-aph (2″) Ia* gene of aminoglycoside-modifying enzymes, and florfenicol/chloramphenicol resistance gene (*cfr*) [116]. Methicillin resistance in *Staphylococcus* is now a global problem [116]. In CNS, the mechanisms of resistance are like those observed in *S. aureus* [116]. However, resistance mediated by the *mecA* gene in CNS is often expressed at lower levels compared to methicillin-resistant *S. aureus* (MRSA) [116]. This lower expression can complicate its detection, highlighting the need for further studies to understand and address these diagnostic challenges [116].

Pyzik et al. [82] analyzed antibiotic resistance in coagulase-negative staphylococci isolated from poultry in Poland. CNS, despite being less pathogenic than coagulase-positive strains, is becoming a significant health threat due to increasing antibiotic resistance [82]. The study detected numerous resistance genes, including the *mecA* gene, suggesting the presence of methicillin-resistant strains of coagulase-negative staphylococci (MR-CNS). Also identified were the *ermA*, *ermB*, and *ermC* genes, which confer resistance to macrolides, lincosamides, and streptogramins, limiting the effectiveness of these antibiotic groups in treating infections. The *tetK* and *tetM* genes, associated with resistance to tetracyclines, were also commonly present, indicating widespread CNS resistance to these frequently used antibiotics in animal treatment. In addition, the study revealed the presence of *blaZ* genes encoding beta-lactamases, which leads to the degradation of beta-lactam antibiotics such as penicillins, further limiting the therapeutic options.

Also, in a study by Chajęcka-Wierzchowska et al., the pheno- and geno-typical antimicrobial resistance profile of CNS from ready-to-eat cured meat was studied [80]. Mainly, *S. epidermidis* and *S. xylosus* were identified. Phenotypic analysis showed that isolates exhibited resistance to FOX, TGC, QD, DA, TET, CN, RD, CIP, W, and SXT, containing the following genes encoding antibiotic resistance in their genome: *mec(A)*, *tet(L)*, *tet(M)*, and *tet(K)*. Notably, two strains of the *S. xylosus* species showed simultaneous antibiotic resistance from nine different classes. This species is a component of the cultures used in the production of meat products, so it also becomes reasonable to control the strains used as starter and protective cultures, which have not been regulated for years and are not mandatorily tested for AMR [117].

A study by Krupa et al. analyzed the antibiotic resistance of *S. aureus* strains isolated from poultry meat in Poland [77]. The study found that a significant percentage of these strains showed resistance to oxacillin, indicating the presence of methicillin-resistant strains of *Staphylococcus aureus* (MRSA). The poultry meat tested in the study also contained MRSA strains, posing a potential risk to consumers. MRSA strains are a serious public health risk due to limited treatment options for infections caused by them [77]. The study observed genotypic diversity in these strains, suggesting multiple sources of infection and transmission between livestock and humans.

Another study by Krupa et al. [78] focused on the population structure and oxacillin resistance in *S. aureus* strains from pork in southwestern Poland. The study found the presence of antibiotic-resistant *S. aureus* strains, including methicillin-resistant *S. aureus*, which exhibit resistance to oxacillin. This resistance is associated with the presence of the *mecA* gene [78]. Other resistance genes such as *erm* (encoding macrolide resistance) and *tet* (encoding tetracycline resistance) were also detected, indicating multidrug resistance in some strains. Phylogenetic analysis revealed a diversity of *S. aureus* clones. Podkowik et al. [81] analyzed in detail the presence of antibiotic-resistant genes in staphylococci isolated from ready-to-eat meat products such as sausages, hams, and pates. The study revealed the presence of numerous resistance genes, suggesting that these products may harbor pathogens resistant to antibiotic treatment. Particular attention was paid to the *mecA* gene. In addition, *erm* genes encoding resistance to macrolides, lincosamides, and streptogramins were detected, further complicating therapy, as these antibiotics are often used to treat staphylococcal infections. *Tet* genes have also been identified that cause resistance to tetracyclines, a group of antibiotics widely used in veterinary medicine and agriculture, suggesting that the use of these drugs in animal husbandry may contribute to the spread of resistant strains in food [81]. The presence of the *blaZ* gene, which encodes beta-lactamases, enzymes that degrade beta-lactam antibiotics (such as penicillins), indicates a wide range of resistance, further limiting treatment options for infections. The study underscores that the high prevalence of these genes in ready-to-eat products poses a real threat to public health, as consumption of contaminated foods can lead to infections that are difficult to treat.

The presence of antibiotic-resistant staphylococci in meat and meat products is a growing food safety concern. The high prevalence of resistance genes and multidrug-resistant strains highlights the need for improved monitoring systems and stricter regulation of antibiotic use in animal husbandry. These findings highlight the necessity of ongoing surveillance of MRSA and other resistant bacteria in animal products to mitigate the risk of transmission to humans and prevent the spread of resistance in the food chain. Additionally, further research is required to better understand resistance mechanisms, develop effective strategies to control them, and address this complex public health issue in the context of food production and processing.

### 5.3. Enterococcus spp.

Enterococci, which are the natural intestinal flora of mammals, birds, and humans, are often responsible for nosocomial infections such as urinary tract infections, endocarditis, and catheter- and wound-related infections [84]. The most frequently isolated species are *Enterococcus faecalis* and *Enterococcus faecium*, whereas *Enterococcus gallinarum* and *Enterococcus casseliflavus* appear less frequently [118]. In poultry, enterococci cause, among others, endocarditis and arthritis [84,118]. The use of antibiotics in human and veterinary medicine promotes the selection of resistant strains, which can transfer resistance genes between different bacteria, posing a risk to human health [119]. In Europe, due to resistance to vancomycin and aminoglycosides, infections caused by enterococci are a serious clinical problem [119]. An example is the use of avoparcin in animal feed, which contributed to the increase in vancomycin resistance before its use was banned in 1997 [120]. Molecular mechanisms of resistance include genes such as *vanA*, *vanB*, *tetM*, or *ermB*, and biofilm-forming enterococci are particularly difficult to control [121]. Biofilms, which are complex communities of microorganisms, protect bacteria from antibiotics and the immune system, making it difficult to treat infections such as wounds or urinary tract infections [84]. The ability to form a biofilm also increases contamination in the food industry and promotes gene transfer between bacteria [84,121].

A study by Chajęcka-Wierzechowska et al. [85] analyzed 390 samples of ready-to-eat meat products, of which *Enterococcus* strains were detected in 74.1%. A total of 302 strains were classified: *E. faecalis* (48.7%), *E. faecium* (39.7%), *E. casseliflavus* (4.3%), *E. durans* (3.0%), *E. hirae* (2.6%), and another *Enterococcus* spp. (1.7%). A high percentage of isolates showed resistance to streptomycin (45.0%), erythromycin (42.7%), fosfomycin (27.2%), rifampicin (19.2%), tetracycline (36.4%), and tigecycline (19.9%). The most frequently detected resistance gene was *ant(6′)-Ia* (79.6%). Other significant genes were *aac(6′)-Ie-aph(2″)-Ia* (18.5%), *aph(3″)-IIIa* (16.6%), and tetracycline resistance genes: *tetM* (43.7%), *tetL* (32.1%), and *tetK* (14.6%). The *ermB* and *ermA* genes were found in 33.8% and 18.9% of isolates, respectively, and almost half of the isolates contained the conjugative transposon Tn916/Tn1545. The study revealed that enterococci are widespread in ready-to-eat meat products. Many of the isolated strains show antibiotic resistance and carry resistance genes that pose a potential risk due to their ability to transmit resistance genes to bacteria present in the human body, which may interact with enterococci isolated from food products. Knowledge of antibiotic resistance in food strains outside the *E. faecalis* and *E. faecium* species is very limited [85]. The experiments conducted in this study analyzed in detail the antibiotic resistance of strains of species such as *E. casseliflavus*, *E. durans*, *E. hirae*, and *E. gallinarum*. The results indicate that these species may also harbor resistance genes to several important classes of antibiotics.

Ławniczek-Wałczyk et al. [83] analyzed the prevalence of antibiotic-resistant *Enterococcus* sp. strains in meat and the production environment of meat plants in Poland. Different *Enterococcus* species were identified, including *E. faecalis* and *E. faecium*. These strains showed significant antibiotic resistance, especially to erythromycin, tetracycline, and vancomycin. Resistance to vancomycin is of particular concern because vancomycin is often the drug of last resort in the treatment of infections caused by multidrug-resistant bacteria. Resistance genes such as *vanA*, *vanB* (for vancomycin), and *ermB* (for erythromycin) are commonly present in strains from both environmental and meat samples.

A study by Stępień-Pyśniak et al. [86] examined the prevalence and antibiotic resistance patterns of *Enterococcus* strains isolated from poultry. It focused on *E. faecalis* and *E. faecium*, which are common in poultry and known for their antibiotic resistance. The results showed that a significant proportion of isolates exhibited multidrug resistance, particularly to antibiotics frequently used in both veterinary and human medicine. High resistance rates were observed for antibiotics such as erythromycin, tetracycline, and vancomycin, with some strains showing resistance to multiple classes of antibiotics.

Woźniak-Biel et al. [84] analyzed the antibiotic resistance of *Enterococcus* strains isolated from turkeys. In the study, 51 strains from turkeys showed high resistance to tetracycline (94.1%) and erythromycin (76.5%). About 43.1% of the strains were multi-resistant, and 15.7% showed vancomycin resistance, associated with the presence of the *vanA* gene. A macrolide resistance gene (*ermB*) was also detected in 68.6% of the strains. All isolates showed the ability to form biofilms, which may contribute to their greater resistance and difficulty in treatment.

The studies presented the widespread occurrence of antibiotic-resistant *Enterococcus* strains in meat and meat products, particularly in ready-to-eat foods and poultry. Multiple studies consistently show that *E. faecalis* and *E. faecium* are the most frequently isolated species, with significant resistance to antibiotics such as tetracycline, erythromycin, and vancomycin. The research points to the frequent presence of antibiotic-resistant genes like *vanA*, *ermB*, *tetM*, and *ermA*. In addition to their high resistance levels, these strains often exhibit the ability to form biofilms, further complicating their treatment and increasing the risk of gene transfer between bacteria. Studies conducted in Poland have revealed that both environmental and meat production facilities are affected by the presence of antibiotic-resistant enterococci, particularly those resistant to clinically important antibiotics like vancomycin, which is often a last-resort treatment. This resistance poses a significant threat to public health by facilitating the transmission of resistant strains through the food chain, from animals to humans.

### 5.4. Listeria monocytogenes

*L. monocytogenes*, a foodborne pathogen that causes listeriosis zoonosis, is increasingly being detected in meat and meat products, raising concerns about food safety and public health. Studies have shown different rates of *L. monocytogenes* in different meats, with chicken, pork, and ready-to-eat meat products being common sources of contamination [118,122,123,124]. The emergence of antibiotic-resistant strains of *L. monocytogenes* in these foods poses a serious threat to human health, as it could compromise the effectiveness of antibiotic therapy for listeriosis [91]. Interestingly, the prevalence and patterns of antibiotic resistance in *L. monocytogenes* isolates from meat and meat products vary across studies and geographic locations [125]. While some studies indicate a relatively low prevalence of antibiotic resistance in *L. monocytogenes* [126], others report a high prevalence of resistant and multidrug-resistant strains [91,125,127]. This discrepancy underscores the need for ongoing monitoring and surveillance of antibiotic resistance in *L. monocytogenes* across regions and food sources.

Kurpas et al. [88] described a detailed genomic analysis of *L. monocytogenes* strains isolated from ready-to-eat meats and surfaces in meat processing plants in Poland. The study identified a variety of *L. monocytogenes* strains that possessed genes encoding resistance to antibiotics from several classes [86]. The *fosB* gene, responsible for resistance to fosfomycin, was detected in several strains. Genes for tetracycline resistance, such as *tetM*, have also been identified. *L. monocytogenes* strains also showed resistance to macrolides due to the presence of the *ermB* gene. Macrolides, such as erythromycin, are often used to treat respiratory and other bacterial infections, and resistance is a major challenge [88]. The study also identified multidrug-resistant strains that simultaneously possessed genes encoding resistance to antibiotics from different classes, including aminoglycosides (e.g., *aacA* gene), β-lactams (e.g., *blaZ* gene), and sulfonamides (e.g., *sul1* gene). These strains have been isolated both from ready-to-eat meat products and from surfaces in processing environments, suggesting that meat processing plants may be a reservoir of antibiotic-resistant strains [86]. The detection of multi-resistant strains in processing environments indicates the possibility of long-term contamination at these sites and the risk of transmission of these strains into meat products [86]. Antibiotic-resistant strains, which can cause severe infections in humans, especially in immunocompromised individuals, pose a serious epidemiological threat [88]. Similar results were reported by Maćkiw et al. [89], who investigated the occurrence and characterization of *L. monocytogenes* in ready-to-eat meat products in Poland. The study revealed the presence of this pathogen in several food samples. *L. monocytogenes* strains were tested for resistance to various antibiotics, and the results showed significant resistance to several key antibiotics. Of most concern was resistance to erythromycin and tetracycline, which are frequently used to treat listeriosis infections. Kawacka et al. [87] present a detailed study on the resistance of *L. monocytogenes* strains isolated from meat products and meat processing environments in Poland. The results showed that most of the analyzed isolates were antibiotic-susceptible to the most-used antibiotics, such as penicillins, macrolides, and tetracyclines, suggesting that current therapies are effective in treating infections associated with food of animal origin [87]. Particular attention was paid to fluoroquinolones, particularly ciprofloxacin, where rare cases of reduced susceptibility were identified, which is worrisome given that fluoroquinolones are key antibiotics in the treatment of many bacterial infections [88]. In contrast, in the study by Skowron et al. [90] assessing the prevalence and antibiotic resistance of *L. monocytogenes* strains isolated from meat, researchers analyzed samples from pork, beef, and poultry over three years. They found that 2.1% of the collected meat samples were contaminated with *L. monocytogenes*, with poultry showing the highest contamination levels. The antibiotic resistance of these strains was concerning, as 6.7% were resistant to all five tested antibiotics. Specifically, the highest resistance rates were observed against cotrimoxazole (45.8%), meropenem (43.3%), erythromycin (40.0%), penicillin (25.8%), and ampicillin (17.5%). Only 32.5% of the strains were sensitive to all antibiotics tested.

The occurrence of *L. monocytogenes* in meat and meat products raises serious food safety and public health concerns, especially due to the emergence of antibiotic-resistant strains. The diversity of prevalence rates and resistance patterns depending on the region and type of product indicates the need for continuous monitoring. Studies in Poland have identified resistance genes to multiple classes of antibiotics, raising concerns about the long-term contamination of meat processing environments and the risk of resistant strains contaminating finished products. Multidrug-resistant strains can significantly hinder the treatment of listeriosis infections, which requires strengthening food safety regulations and further research into resistance mechanisms. Furthermore, the findings emphasize the importance of microbiological monitoring and control in meat processing plants to prevent the spread of resistant *L. monocytogenes*. Regular research into antibiotic resistance among food-related pathogens is crucial, alongside the implementation of appropriate control procedures in food production. Ultimately, further research into resistance mechanisms and their implications is needed to better protect public health.

### 5.5. Enterobacterales

The annual report on trends and sources of zoonoses published in December 2021 by the European Food Safety Authority (EFSA) and the European Center for Disease Prevention and Control (ECDC) shows that nearly one in four foodborne outbreaks in the European Union (EU) in 2020 were caused by *Salmonella* spp., making this bacterium the most reported causative agent of foodborne outbreaks (694 foodborne outbreaks in 2020) [128]. *Salmonella* spp. infections in humans are usually caused by the consumption of food of animal origin, mainly eggs, poultry, or pork [129,130]. An analysis by Gutema et al. [129] show that beef and veal can also be a source of *Salmonella* spp. infection because these animals are potential asymptomatic carriers. Multidrug-resistant *Salmonella* poses a serious threat to public health after foodborne infections [131]. Today, such multidrug-resistant strains are increasingly being isolated from beef, pork [132,133], and poultry [134]. According to the monitoring of antimicrobial resistance in food and food-producing bacteria, as specified in Commission Implementing Decision 2013/652/EU, *Salmonella* antibiotic resistance isolated from food and food-producing animals should target broilers, fattening pigs, calves under one year old, and their meat [135].

A study by Szewczyk et al. [92] on the antibiotic resistance of Enterobacterales strains isolated from food showed that most strains (28.0–65.1%) were resistant to a single antibiotic, but 15 strains (34.9%) were resistant to two or more antibiotics. Particularly prominent among them were strains of *Escherichia coli* and *Proteus mirabilis*, which were resistant to multiple antibiotics, including beta-lactams (piperacillin, cefuroxime, and cefotaxime), fluoroquinolones, and carbapenems. All isolates were sensitive to gentamicin, and none showed ESBL-type resistance. Strains resistant to high concentrations of antibiotics (256 μg/mL) included *Salmonella* spp., *Hafnia alvei*, *P. mirabilis*, and *E. coli*. Beta-lactamase-resistant and piperacillin- and cefuroxime-resistant *Klebsiella* strains (including *K. ozaenae* and *K. rhinoscleromatis*) suggested the ability to produce beta-lactamase enzymes (AmpC and CTX-M), which allows resistance transfer between species.

Zarzecka et al. [94] examined in detail the incidence of antibiotic resistance in Enterobacterales strains isolated from raw meat and ready-to-eat meat products. The highest number of isolated strains was identified as *E. cloacae* (42.4%), followed by *E. coli* (9.8%), *P. mirabilis*, *S. enterica*, *P. penneri*, and *C. freundii* (7.6% each), and *C. braakii* (6.6%), *K. pneumoniae*, and *K. oxytoca* (5.4% each). More than half of the isolated strains (52.2%) showed resistance to at least one antibiotic, with the highest number of resistant strains found against amoxicillin with clavulanic acid (28.3%) and ampicillin (19.5%). The ESBL (+) phenotype was found in 26 strains, while the AmpC (+) phenotype was found in 32 strains. The *bla*CTX-M gene was present in 53.8% of the ESBL-positive strains, and the CIT family gene was present in 43.8% of the AmpC-positive strains [94]. Raw meat has been identified as a key source of resistant strains, posing a significant public health risk, especially in the context of ready-to-eat products, which can be exposed to improper processing, lack of proper sanitary–epidemiological control and improper storage [94]. Both phenotypic analyses, such as antibiotic susceptibility tests, and genotypic analyses were used in the study, which made it possible to accurately determine the resistance profiles of the tested strains.

Mąka et al. [63] analyzed the antibiotic resistance profiles of *Salmonella* strains isolated from retail meat products in Poland between 2008 and 2012. The results of the study showed that more than 90.0% of the strains exhibited resistance to at least one antibiotic, indicating a high level of resistance in the bacterial population. The highest resistance was found against tetracycline, streptomycin, and sulfonamides, reflecting the widespread use of these antibiotics in animal husbandry. Strains of *S. typhimurium* were more resistant than other serotypes, with about 20.0% of them showing resistance to five or more classes of antibiotics, classifying them as multi-resistant. Resistance to fluoroquinolones, which are often used to treat *Salmonella* sp. infections in humans, was also found.

In a study by Pławińska-Czernak et al. [96], researchers analyzed the occurrence of multidrug resistance in *Salmonella* strains isolated from raw meat products such as poultry, beef, and pork. The study showed that 64.3% of the isolates showed resistance to at least three classes of antibiotics, with the highest resistance reported against tetracyclines (56.5%), aminoglycosides (47.8%), beta-lactams (34.8%), and quinolones (30.4%). A key aspect of the study was the identification of genes encoding resistance, including the *tetA*, *blaTEM*, *aadA*, and *qnrS* genes, which were responsible for resistance to tetracyclines, beta-lactams, aminoglycosides, and quinolones, respectively. The presence of these genes indicates the widespread spread of genetic resistance among food-related pathogens, which poses a serious threat to public health.

Sarowska et al. [93] examined the antibiotic resistance and pathogenicity of *E. coli* strains from poultry farms, retail meat, and human urinary tract infections. The strains showed significant resistance to a variety of antibiotic classes, including β-lactams, tetracyclines, aminoglycosides, fluoroquinolones, and sulfonamides, indicating the widespread selection pressure exerted by antibiotic use in poultry farming. *E. coli* strains from meat and poultry farms showed some commonalities with isolates causing human infections, suggesting the possibility that potentially pathogenic strains could be transmitted through the food chain.

In the presented studies, the researchers highlight the urgent need for continuous monitoring of antibiotic resistance in animal products, along with the implementation of stricter sanitary standards in the food industry. The researchers emphasize educating producers and consumers about the risks of antibiotic resistance to minimize the risk of foodborne infections. Considering the changing resistance profiles, the researchers recommend regular monitoring and restriction of antibiotic use in agriculture, supported by stricter regulations to prevent the spread of resistant strains, especially *Salmonella*. Multidrug-resistant strains of *Salmonella*, which are increasingly resistant to tetracyclines, aminoglycosides, and beta-lactams, pose a serious threat to public health. Similarly, high levels of antibiotic resistance have been observed in Enterobacterales strains, including *E. coli*, isolated from raw meat and animal products. Particular attention was paid to ESBL (+) and AmpC (+) strains, highlighting the importance of reducing antibiotic use in animal husbandry and strengthening sanitary controls in meat processing. The study also highlights the importance of monitoring food safety and zoonotic infection risks to reduce the spread of multidrug-resistant pathogens via food.

## 6. Alternatives to Antibiotic Therapy in Agriculture and Animal Husbandry

**Figure 2:**
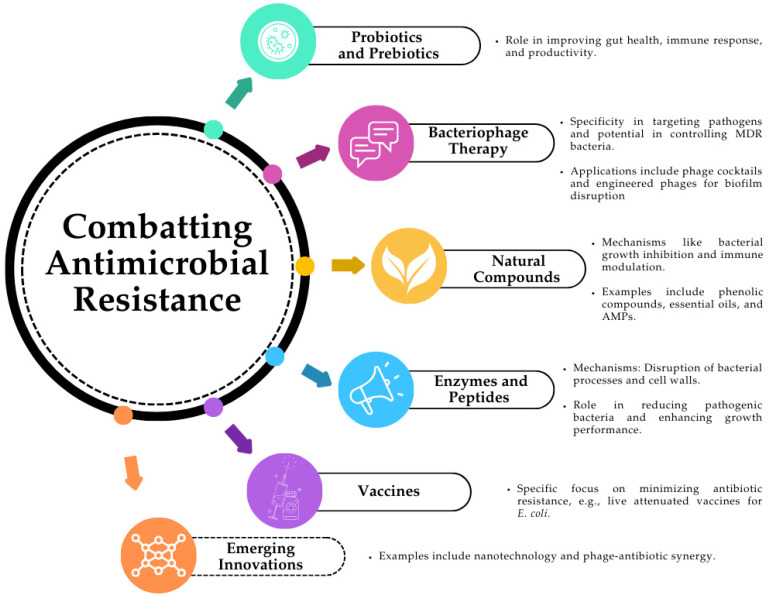
Alternatives to antibiotic therapy in agriculture and animal husbandry.

Alternatives to antibiotic therapy in agriculture and animal husbandry are increasingly being explored to combat the rising challenge of antimicrobial resistance and the negative environmental impacts of excessive antibiotic use [136] (Figure 2).

### 6.1. Probiotics and Prebiotics

Probiotics and prebiotics represent a promising alternative [137]. Probiotics are live microorganisms, typically beneficial bacteria, which confer health benefits to the host when administered adequately [138]. Several health and nutritional benefits have been observed to be provided to animals by probiotics. They promote animal growth and maturation [139] and increase feed intake, digestibility, and performance [137,140]. Other benefits include improved health outcomes and immune responses [141], egg production [142], meat yield and its quality [138,143,144], and milk composition and its production in ruminants [145,146]. In turn, prebiotics are compounds that induce the growth or activity of beneficial microorganisms, particularly in the gut [147]. When used together, as symbiotics, they promote gut health by enhancing the balance of gut microbiota, which is crucial for maintaining the immune system’s strength [147]. According to Low et al. [147], these supplements can enhance animal health, improve feed efficiency, and boost growth without relying on antibiotics. This approach is particularly promising in preventing intestinal infections and supporting overall gut immunity, thereby reducing the need for antibiotic interventions. Gupta et al. [148] suggested that symbiotics can help mitigate the need for antibiotics by boosting the animal’s natural defenses against infections. This dual approach is seen as an effective way to improve productivity and animal welfare without the overuse of antibiotics, particularly in poultry and swine production. Śmiałek et al. [149] used a multispecies probiotic (Lavipan, JHJ, Poland) containing *Lactococcus lactis*, *Carnobacterium divergens*, *Lactiplantibacillus casei*, *Lactiplantibacillus plantarum*, and *Saccharomyces cerevisiae* in broiler feeding to effectively reduce contamination of poultry with *Campylobacter* spp. The use of the probiotic reduced colonization of the chickens’ digestive tract and reduced environmental and poultry carcass contamination. In addition, the probiotic supported the poultry’s immune system, improving carcass hygiene parameters and reducing the risk of pathogen transmission in the food chain. The results of the presented research highlight the potential of probiotics as an alternative to antibiotics in poultry farming, supporting sustainable agricultural practices and food safety [149]. Future research should focus on multi-strain probiotic formulations tailored to specific livestock species and regional conditions. Advances in genetic engineering could lead to probiotics with enhanced functionalities, such as targeted pathogen inhibition or increased gut resilience [147].

### 6.2. Bacteriophages

Bacteriophages (phages) are emerging as an innovative and natural alternative to traditional antibiotics, particularly in the battle against multidrug-resistant (MDR) bacteria. These viruses specifically infect and lyse bacterial cells, with a high degree of host specificity, making them valuable tools for targeting pathogenic bacteria without disrupting beneficial microbiota [136]. In agriculture, phages are being explored for controlling bacterial infections in livestock and crops, offering environmentally friendly solutions. They can be administered via water, feed, or directly to infected plants or animals, making them versatile agents in sustainable farming systems [150]. Recent advancements include genetically engineered phages and phage-derived enzymes like lysins, which significantly enhance antibacterial efficacy by breaking down bacterial cell walls. Such innovations have shown promise not only in agriculture but also in clinical settings for wound care and biofilm eradication, where MDR pathogens pose severe threats [151]. Phage–antibiotic synergy (PAS) is another area of growing interest, where the combination of phages and sub-lethal doses of antibiotics enhances bacterial clearance while reducing the likelihood of resistance development [152]. Phage therapy’s specificity is particularly advantageous in addressing biofilms, which are notoriously resistant to antibiotics. Phage cocktails, designed to target multiple bacterial strains, have shown substantial efficacy in disrupting biofilms in healthcare settings [153]. Additionally, bacteriophages offer a unique potential for antivirulence strategies, where phage-induced bacterial resistance may simultaneously reduce bacterial fitness and virulence, further attenuating infections [154]. Despite their vast potential, challenges persist. Regulatory barriers, the need for standardized safety profiles, and the risk of phage resistance require further research and policy development [155]. Nevertheless, with advancements in genetic engineering and better understanding of phage biology, bacteriophages hold immense promise as versatile and sustainable alternatives to antibiotics in diverse applications.

### 6.3. Natural Compounds

Natural compounds play a pivotal role in addressing the global challenge of antimicrobial resistance (AMR), as demonstrated by their diverse mechanisms of action and potential benefits widely discussed in the scientific literature. The use of natural compounds in combating antibiotic resistance is widely discussed in the scientific literature, demonstrating their various mechanisms of action and potential benefits. For example, polyphenolic compounds such as curcumin, resveratrol, and gallic acid can act as photosensitizers in photodynamic therapy, effectively destroying bacterial biofilms and aiding in the treatment of infections [156]. Marine-derived products, on the other hand, offer unique chemical structures that can be effective against multidrug-resistant bacteria [157].

Phytogenic compounds derived from medicinal plants, including essential oils, alkaloids, and phenolic compounds, have gained traction for their antimicrobial, antioxidant, and anti-inflammatory properties. These plant-based alternatives include essential oils, alkaloids, and phenolic compounds, which possess antimicrobial, antioxidant, and anti-inflammatory properties. Gao et al. [158] explained that phenolic compounds from medicinal plants can inhibit bacterial growth and modulate the gut microbiome in animals, thus supporting health and growth. These natural extracts are also being studied for their role in enhancing the animal immune system, which further reduces the need for antibiotics [158]. Phytogenic is seen as a sustainable alternative that can improve both animal welfare and productivity. The use of phytotherapeutics has pointed to their bactericidal properties and ability to reverse drug resistance, although challenges such as overexploitation of resources and climate impacts limit their wider use [159].

Another innovative approach involves essential oils (EOs), which show multifaceted bactericidal activity and potential as coatings in me-too devices to prevent infections, highlighting their versatility and efficacy compared to synthetic antibiotics [160]. Moreover, molecular docking studies of plant-derived compounds against specific pathogenic targets illustrate their untapped potential in combating protozoan and bacterial resistance [160]. Plant extracts and secondary metabolites, such as terpenoids or alkaloids, also show promising antimicrobial activity, as detailed in reviews of their use as bioactive food preservatives and potential therapeutic candidates [161].

### 6.4. Enzymes and Peptides

Another promising approach is the use of ribosomal antimicrobial peptides (AMPs), which disrupt bacterial processes and serve as a potential alternative to conventional antibiotics [162]. AMPs are known for their multifunctional role in disrupting bacterial processes, offering a promising alternative to conventional antibiotics [162]. AMPs, along with enzymes like lysozymes, can be incorporated into animal feed to reduce pathogenic bacteria in the gut and improve growth performance while avoiding resistance development [156,163]. Enzymes and antimicrobial peptides also show great potential as alternatives to antibiotics. Enzymes, such as proteases and lysozymes, help break down microbial cell walls, while AMPs are small proteins found naturally in many organisms that exhibit broad-spectrum antimicrobial activity. Wang et al. [156] emphasized that these compounds can be incorporated into animal feed to reduce pathogenic bacteria in the gut and improve the overall growth performance of animals. Synthetic AMPs offer a natural, non-toxic method of reducing pathogen loads without leading to resistance, making them an ideal candidate for replacing antibiotics in animal production systems [163]. Zhang et al. [163] highlighted synthetic AMPs as a promising advancement, combining stability with cost-effectiveness.

In addition, natural products such as antimicrobial peptides and fungal-derived compounds offer new opportunities to modulate multidrug resistance [163]. It is also important to consider biotechnological modifications of natural sources to increase their availability and effectiveness [164]. Research on nano-antioxidants and phage therapy as additional methods to combat AMR is also groundbreaking [165]. The past successes of naturally derived antibiotics underscore the importance of integrating traditional knowledge with modern research methods [160]. All this evidence points to the crucial role of natural products in the development of future antimicrobial therapies.

### 6.5. Vaccines

The research also observes the design of vaccines with the specific purpose of minimizing antibiotic resistance for specific groups of microorganisms. Śmiałek et al. [166] indicated that the use of a live attenuated vaccine against *E. coli* can effectively reduce the use of antibiotics in broiler breeding. The use of the vaccine showed a significant reduction in the number of multi-resistant *E. coli* strains, increasing their sensitivity to antibiotics. At the same time, vaccinated broilers showed better production parameters, such as faster weight gain and lower mortality, and the vaccination did not adversely affect the effectiveness of other vaccines. The results suggest that the routine use of *E. coli* vaccine in immunoprophylaxis programs can help improve flock health, reduce the risk of antibiotic resistance, and improve production performance, which is crucial for sustainable poultry farming management [166].

### 6.6. Emerging Innovations

One innovative solution is the use of nanoparticles (NPs), which exhibit antibacterial properties, raising hopes for their use in the fight against drug-resistant pathogens [8]. Thanks to their properties, they not only have antibacterial effects themselves, but can also be carriers for antibiotics and natural antimicrobial compounds [8]. Examples of such nanoparticles include Ag-NP, Zn-NP, Au-NP, Al-NP, Cu-NP, and Ti-NP, and metal oxide nanoparticles such as ZnO-NP, CdO-NP, CuO-NP, and TiO_2_-NP, among others. All these structures have shown effectiveness in destroying bacteria [167].

A study by Joost et al. [168] confirmed that treatment with TiO_2_ nanoparticles can lead to an increase in the volume of bacterial cells, causing damage to their cell membranes and death. They have also been shown to be effective against multidrug-resistant (MDR) pathogens such as *E. coli*, *K. pneumoniae*, *Pseudomonas aeruginosa*, *Acinetobacter baumannii*, methicillin-resistant *S. aureus*, and *E. faecalis*. The mechanism involves the generation of reactive oxygen species (ROS), which leads to oxidative stress in pathogen cells [169,170,171].

Nanoparticles are also being explored as carriers for antibiotics to increase the effectiveness of therapy and minimize the risk of developing bacterial resistance [172]. The conjugation of antibiotics, such as ampicillin, kanamycin, or streptomycin, with gold NPs has achieved lower minimum inhibitory concentrations against Gram-positive and Gram-negative bacteria than with the drugs used alone [173]. Similarly, vancomycin-loaded gold nanoparticles showed enhanced efficacy against strains resistant to this antibiotic by disrupting the stability of bacterial cell membranes [174].

Studies have also shown that bimetallic nanoparticles, such as combinations of two different metals, are more effective than their monometallic counterparts [175,176,177]. They have better electron, optical, and catalytic properties, which translates into many times greater efficacy against MDR pathogens while reducing the required therapeutic dose [175,176,177]

The growing focus on alternatives to antibiotics in agriculture and animal husbandry is a response to the urgent need to combat AMR and reduce the environmental footprint of traditional farming practices. Probiotics, prebiotics, vaccines, phage therapy, medicinal plant extracts, enzymes, and antimicrobial peptides all represent promising tools in this effort. These strategies help maintain animal health, improve productivity, and reduce dependency on antibiotics, thus offering a sustainable path forward for the agricultural industry.

## 7. Conclusions

Antimicrobial resistance in meat and meat products in Poland presents several challenges for public health, food safety, and environmental sustainability that require a more critical and coordinated approach. In Poland, the increasing prevalence of antibiotic-resistant bacteria in meat and meat products underscores the critical need for effective strategies to mitigate the spread of resistance. Microorganisms such as *Campylobacter* spp., *Staphylococcus* spp., *Enterococcus* spp., *L. monocytogenes*, and Enterobacterales (including *Salmonella* spp. and *E. coli*) are commonly found in animal farming environments and food products, often exhibiting resistance to multiple classes of antibiotics. Current data on AMR are limited to isolated studies, with a lack of comprehensive nationwide surveillance, which hampers our understanding of resistance patterns across different regions and food products. The cited research results highlight the critical need for a multifaceted approach to antimicrobial resistance management in Poland, including stricter controls on antibiotic use in animal husbandry, improved monitoring of resistance patterns and the promotion of alternative strategies to reduce antibiotic dependence. Additionally, inconsistent application of monitoring systems and weak regulatory enforcement on antibiotic usage in livestock production contribute to the persistence of AMR. The environmental impact of farming practices, particularly the contamination of soil and water with resistant bacteria and genes, remains under-researched but is likely a significant pathway for the spread of AMR. To address these issues, future efforts must focus on establishing a standardized, nationwide surveillance system for monitoring both antibiotic usage and resistance in livestock. Moreover, further research is needed to understand the environmental persistence of AMR, particularly in regions with intensive farming operations. There is also a growing need for alternatives to antibiotics, such as probiotics, phage therapy, and antimicrobial peptides, to reduce dependency on traditional antibiotics in agriculture. Strengthening regulatory frameworks, improving compliance with EU standards, and raising awareness about the risks of AMR among farmers and veterinarians will be crucial. By focusing on these areas, Poland can make significant progress in controlling the spread of AMR in its food systems and protecting public health and the environment.

## Figures and Tables

**Table 1:** Different food processing techniques influence antibiotic content changes in different meat.

Processing	Product Type	Parameters	Antibiotic	Initial Value [μg/kg]	Value After Heat Treatment [μg/kg]	Reduction [%]	Reference
**Boiling**	Chicken	100 °C/5 min	ENO	746.34 ± 5.62	237.53 ± 2.13	68.17	[46]
			OTC	824.16 ± 7.20	383.33 ± 3.70	53.49	
			DOX	680.84 ± 8.84	425.53 ± 5.65	37.50	
			CIP	643.14 ± 6.97	205.46 ± 9.72	68.05	
		100 °C/2 min	TET	100,000	43,898 ± 2362	56.10	[40]
	Pork				42,332 ± 2881	57.67	
	Chicken	100 °C/3 min	OTC	500	365.95 ± 6.84	26.81	[42]
		100 °C/6 min			325.71 ± 4.92	34.86	
		100 °C/15 min			249.13 ± 4.89	50.17	
	Pork	100 °C/3 min			360.09 ± 3.65	27.98	
		100 °C/6 min			317.08 ± 4.17	36.58	
		100 °C/15 min			236.56 ± 7.96	52.69	
	Chicken	100 °C/3 min	SDZ	84	50	40.48	[44]
			SMX	252	172	31.75	
			SMM	436	315	27.75	
			SQ	970	669	31.03	
		100 °C/6 min	SDZ	84	41	51.19	
			SMX	252	145	42.46	
			SMM	436	274	37.16	
			SQ	970	584	39.79	
		100 °C/9 min	SDZ	84	34	59.52	
			SMX	252	129	48.81	
			SMM	436	248	43.12	
			SQ	970	539	44.43	
		100 °C/12 min	SDZ	84	33	60.71	
			SMX	252	117	53.57	
			SMM	436	239	45.18	
			SQ	970	518	46.60	
**Roasting**	Chicken	200 °C/30 min	ENO	746.34 ± 5.62	233.23 ± 10.19	68.75	[46]
			OTC	824.16 ± 7.20	274.72 ± 3.40	66.67	
			DOX	680.84 ± 8.84	340.42 ± 4.92	50.00	
			CIP	643.14 ± 6.97	200.98 ± 10.02	68.75	
		170 °C/3 min	SDZ	82	77	6.10	[44]
			SMX	322	301	6.52	
			SMM	560	462	17.50	
			SQ	1145	1005	12.23	
		170 °C/6 min	SDZ	82	80	2.44	
			SMX	322	271	15.84	
			SMM	560	420	25.00	
			SQ	1145	897	21.66	
		170 °C/9 min	SDZ	82	80	2.44	
			SMX	322	255	20.81	
			SMM	560	400	28.57	
			SQ	1145	851	25.68	
		170 °C/12 min	SDZ	82	79	3.66	
			SMX	322	198	38.51	
			SMM	560	337	39.82	
			SQ	1145	713	37.73	
**Microwave** **cooking**	Chicken	900 W/3 min	ENO	746.34 ± 5.62	334.68 ± 3.63	55.16	[46]
			OTC	824.16 ± 7.20	227.67 ± 2.10	72.38	
			DOX	680.84 ± 8.84	544.67 ± 6.67	20.00	
			CIP	643.14 ± 6.97	288.40 ± 3.23	55.16	
		440 W/0.75 min	TET	100,000	40,111 ± 13,979	59.89	[40]
	Pork				19,463 ± 2652	80.54	
	Chicken	800 W/0.5 min	OTC	500	342.18 ± 5.32	31.56	[42]
		800 W/1 min			275.69 ± 3.21	44.86	
		800 W/2 min			223.56 ± 4.45	55.29	
	Pork	800 W/0.5 min			355.82 ± 1.71	28.84	
		800 W/1 min			309.07 ± 0.72	38.19	
		800 W/2 min			204.75 ± 1.17	59.05	
**Grilling**	Chicken	8 kW/2.5 min	ENO	746.34 ± 5.62	497.56 ± 4.75	33.33	[46]
			OTC	824.16 ± 7.20	686.80 ± 6.50	16.67	
			DOX	680.84 ± 8.84	567.37 ± 6.20	16.66	
			CIP	643.14 ± 6.97	535.95 ± 5.31	16.67	

Abbreviations: ENO—enrofloxacin; CIP—ciprofloxacin; DOX—doxycycline; OTC—oxytetracycline; SDZ—sulfadiazine; SMX—sulfamethoxazole; SMM—sulfamonomethoxine; SQ—sulfaquinoxaline; TET—tetracycline.

**Table 2:** Occurrence and antimicrobial resistance of microorganisms isolated from meat and meat products in Poland.

No.	Microorganisms	Product Type	Antimicrobial Resistance			References
			Methods for Detecting Antimicrobial Resistance	Antimicrobial Resistance Tested	Resistance Genes Tested	
1	*C. jejuni*, *C. coli*	Beef and pork (raw meat)	Disk diffusion	CIP, E, CN, TET, AZM	NA	[69]
2		Bovine and pork carcasses	Microbroth dilution	CN, C, NAL, CIP, STR, E, TET		[70]
3		Raw chicken meat (wings, legs, carcass frames, filets, and ground meat) and offal (livers, hearts, and gizzards)	Disk diffusion	TET, CIP, E		[71]
4		Chicken broiler carcasses		E, CIP, CN, NAL, STR, TET		[72]
5		Poultry broiler carcasses	Microbroth dilution	CIP, TET, E		[73]
6		Turkey and broiler carcasses	Microbroth dilution and PCR assay	AZM, CIP, E, CN, TET, FLR, NAL, TEL, DA	*gyrA*, *tetO*, *cmeB*	[74]
7		Domestic geese	Disk diffusion	E, CN, CIP, AMP, TET, C, NAL	NA	[75]
8	*C. jejuni*, *C. coli*, *Campylobacter* spp.	Raw chicken meat from wings, legs, corpuses, filets, ground meat, and offal samples (livers, hearts, and gizzards)	Disk diffusion and PCR assay	CIP, TET, E, CN	*gyrA*, *tetO*	[76]
9	*S. aureus*	Chicken meat samples (legs and wings)		P, CE, TET, DA, CN, E, OXA	*mecA*, *mecC*, *blaZ*	[77]
10		Samples of pork meat from company shops		P, TET, DA, CN, E, CIP, NOR, VA	*blaZ*	[78]
11	*S. aureus*, *S. xylosus*	Cured meat		CE, TGC, QD, DA, TET, CN, RD, CIP, W, SXT	*mec(A)*, *tet(L)*, *tet(M)*, *tet(K)*	[79,80]
12	*S. aureus*, CNS (*S. xylosus*, *S. epidermidis*, *S. xylosus*), *Staphylococcus* spp.	Sausage		DA, CE, F, TGC, SXT, C, RD, CN, LZD, E, NOR, W, CIP, QD, TET		
13	*S. epidermidis*	Poultry		DA, CE, LZD, QD	*mec(A)*, *tet(M)*, *tet(K)*	
14	*S. aureus*, CNS (*S. epidermidis*, *S. pasteuri*, *S. haemolyticus*, *S. carnosus*, *S. saprophyticus*, *S. sciuri*, *S. chromogenes*, *S. capitis*, *S. xylosus*, *S. equorum*, *S. lugdunensis*)	Ready-to-eat meat products		OXA, P, TET, E, CN, VA	*mecA*, *blaZ*, *tetO/K/M*, *ermA/B/C*, *aph*, *vanA/B/C/D*	[81]
15	CNS (*S. cohnii*, *S. epidermidis*, *S. haemolyticus*, *S. hominis*, *S. simulans*, *S. saprophyticus*, *S. lentus*, *S. xylosus*, *S. sciuri*, *S. chromogenes*)	Broiler chickens and turkeys		AMX/CL, AMX, AMP, P, CE, DA, C, E, CN, TET, SXT	*blaZ*, *mecA*, *aac(6′)-aph(2″)*, *ermA*, *ermB*, *msrA/B*, *tetM*, *tetK*, *tetL*, *tetO*, *cfr*	[82]
16	*E. faecalis*, *E. faecium*	Raw pork meat		E, TET, VA	*ermB*, *vanA*, *vanB*	[83]
17	*E. faecalis*, *E. faceium*, *E. gallinarum*	Turkeys	Disk diffusion and Multiplex PCR assay	AMP, AMX/CL, VA, CIP, TET, E, CN	*blaZ*, *vanA*, *vanB*, *vanC-1*, *tetK*, *tetM*, *tetO*, *ermA*, *ermB*, *ermC*, *aac(6′)Ie-aph(2″)Ia*	[84]
18	*E. faecalis*, *E. faecium*, *E. casseliflavus*, *E. durans*, *E. hirae*, *Enterococcus* spp.	Ready-to-eat meat products: smoked meat (ham, shoulder, bacon, and tenderloin); sausages (simmered and boiled); offal products (liver sausage, blood sausage, and brawn); formed meat products; tinned products (meat, offal products, and terrine)	Disk diffusion and PCR assay	AMP, PM, CN, STR, TEC, NOR, LEV, CIP, TET, TGC, RD, F, LZD, FOS, C, QD, E	*aac(6′)-le-aph (2‴)-la*, *aph (2″)-Ib*, *aph(2‴)-1c*, *aph (2″) -Id*, *aph(3″)-Illa*, *ant (4′)-la*, *ant (6′)-la*, *tetM*, *tetL*, *tetK*, *tetO*, *ermA*, *ermB*, *ermC*, *msrC*, *mefA/E*, *vanC2/C3*	[85]
19	*E. cecorum*, *E. faecalis*, *E. faecium*, *E. hirae*, *E. gallinarum*, *E. casseliflavus*, *E. avium*, *E. columbae*	Hearts, livers, brains, bone marrow, and oviduct swabs from poultry	Disk diffusion	VA, AMX, AMX/CL, DOX, E, FLR, LIN/SP, TY, SXT	NA	[86]
20	*L. monocytogenes*	Meat food samples (raw and processed) and meat processing environment (both contacting and non-contacting with food)		AMP, C, E, CN, P, STR, SXT, TET, VA, CIP		[87]
21		Different kinds of ready-to-eat (RTE) foods of animal origin (e.g., ham, sausages, or meat)	Genotypic data–BIGSdb-Lm platform (Institut Pasteur, Paris, France)	NA	*fosB*, *tetM*, *ermB*, *aacA*, *blaZ*, *sulI*	[88]
22		RTE meat and meat product samples (Dumplings with meat, chicken cutlet, chicken gyros, chicken in jelly, pork in jelly, chicken salad, rice with meat, roll with chicken gyros and vegetables, steak tartare (raw beef), pork stew, chicken shish kebab, poultry meat, beef meat, smoked poultry sausage, headcheese, luncheon meat, chicken paste, mett (raw sausage), roasted pork loin, cooked pork ham, polish type sausage, and pâté with boletus	Disk diffusion	CN, MEM, AMP, SXT, AMX/CL, C, CIP, E, TET	NA	[89]
23		Various types of meat (pork, beef, and poultry)		P, AMP, MEM, E, SXT		[90]
24		Chicken breast filet	Disk diffusion and MTS^TM^ (MIC Test Strips) (Liofilchem^®^, Roseto degli Abruzzi, TE, Italy)	DA		[91]
25	Enterobacterales: *Escherichia* sp., *Klebsiella* sp., *Serratia* sp., *Enterobacter* sp., *Proteus* sp., *Hafnia* sp., *Citrobacter* sp., *Salmonella* sp., *Shigella* sp.	Samples of fresh raw meat (poultry, pork, beef, and mechanically minced meat) and processed meat (cured meats) intended for sale, obtained from meat processing plants	ETEST^®^ (BioMérieux, Craponne, France)	CFU, PIP, NAL, CIP, CAZ, CTX, SXT, IMP, TOB, PIP/TAZ		[92]
26	*E. coli*	Raw meat (chicken, turkey, pork, and beef)	Disk diffusion	AMX, TET, SXT, CIP, PIP, NIT, C, AMX/CL, CFT, CN, PIP/TAZ, CE, CAZ, MEM, IMP, AMK		[93]
27	*E. cloacae*, *S. enterica*, *P. penneri*, *C. braakii*, *P. penneri* *P. mirabilis*, *K. oxytoca*, *E. coli*, *C. braakii*, *C. freundii*, *K. pneumoniae*	Ready-to-eat foods of animal origin (sausages, bacon, pate, gammon, brawn, salami, and roasted meat) and raw meat (beef, poultry, pork, and veal)	ESBL + AmpC screen disk kit (Liofilchem^®^, Roseto degli Abruzzi, TE, Italy) and PCR assay	AMP, PIP, AMX/CL, CTX, CAZ, IMP, CN, TOB, LEV, TET, SXT	*Bla_CTX-M_*, *bla_TEM_*_,_*bla_oxa_*, *bla_SHV_*, *ACC*, *mox*, *dha*, *cit*, *ebc*, *fox*, *tet(M)*, *tet(L)*, *tet(K)*, *aac(6′)-Ii*, *ant(6)-Ia*, *aac(6ʹ)-Ie-aph(2″)-Ia*, *aph(3ʹ)-IIIa*	[94]
28	*S. enteritidis*, *S. infantis*, *S. typhimurium*	Retail meat product samples: poultry, meat, pork, beef, and mixed meat	Disk diffusion	NAL, TET, AMP, STR, SUL	NA	[95]
29	*S. enteritidis*, *S. infantis*, *S. typhimurium*, *S. hadar*, *S. newport*, *S. virchow*, *S. chester*, *S. agonal*, *S. saintpaul*, *S. derby*, *S. duisburg*, *S. sandiego*, *S. anatum*, *S. brandenburg*, *S. eko*, *S. glostrup*, *S. heidelberg*, *S. indiana*, *S. kottbus*, *S. mbandaka*, *S. wippra*	Poultry meat, pork, beef, and mixed meat		ATM, AMX/CL, AMP, CE, C, CN, NAL, SUL, STR, TET, W, SXT		[63]
30	*S. enterica* spp. *Enterica*, *S. enteritidis* *S. infantis*, *S. newport*, *S. derby*, *S. indiana*, *S. mbandaka*, *S. kentucky*	Pork and poultry sample of meat	VITEK^®^ 2 System and AST-GN96 cards for Gramnegative bacteria (BioMérieux, Craponne, France), microdilution, PCR assay	AMP, AMX, AMX/CL, CFX, CFT, CPH, CFX, CFTI, CFQ, IMP, CN, NEO, STR, ENR, UB, MRB, NOR, DOX, OXY, TET, FLR, LIN/SP, SXT	*bla_CMY-2_*, *bla_PSE-1_*, *bla_TEM_*, *aadA*, *aadB*, *strA/strB*, *floR*, *dfrA1*, *dfrA12*, *sul1*, *sul2*, *sul3*, *bla_SHV_*, *aphA1*, *aphA2*, *tetA*, *tetB*, *bla_PSE-1_*	[96]

Abbreviations: CNS—coagulase-negative staphylococci; NA—not analyzed; PCR—polymerase chain reaction; AMP—ampicillin; AMX—amoxicillin; AMX/CL—amoxicillin and clavulanic acid; ATM—aztreonam; AZM—azithromycin; C—chloramphenicol; CAZ—ceftazidime; CE—cefoxitin; CFT—cephalothin; CPH—cefapirin; CFU—cefuroxime; CFQ—cefquinome; CFTI—ceftiofur; CFX—cephalexin; CIP—ciprofloxacin; CN—gentamycin; CTX—cefotaxime; DA—clindamycin; DOX—doxycycline; E—erythromycin; ENR—enrofloxacin; F—nitrofurantoina; FLR—florfenicol; UB—flumequine; FOS—fosfomycin; IMP—imipenem; LEV—levofloxacin; LIN/SP—lincomycin/spectinomycin; LZD—linezolid; MEM—meropenem; MRB—marbofloxacin; NAL—nalixid acid; NEO—neomycin; NIT—nitrofurantoin; NOR—norfloxacin; OXA—oxacillin; OXY—oxytetracycline; P—penicillin; PM—polymyxin; PIP—piperacillin; PIP/TAZ—piperacillin/tazobactam; QD—quinupristin/dalfopristin; RD—rifampicin; STR—streptomycin; SUL—sulphoamides comp.; SXT—trimethoprim-sulfamethoxazole; TEC—teicoplanin; TOB—tobramycin; TEL—telithromycin; TET—tetracycline; TGC—tigecycline; TY—tylosin; VA—vancomycin; W—trimethoprim.

## Data Availability

Data are contained within the article.
